# Comparative Evaluation of Cytotoxic and Apoptogenic Effects of Several Coumarins on Human Cancer Cell Lines: Osthole Induces Apoptosis in p53-Deficient H1299 Cells

**DOI:** 10.1155/2014/847574

**Published:** 2014-09-03

**Authors:** Yalda Shokoohinia, Leila Hosseinzadeh, Maryam Alipour, Ali Mostafaie, Hamid-Reza Mohammadi-Motlagh

**Affiliations:** ^1^Novel Drug Delivery Research Center, School of Pharmacy, Kermanshah University of Medical Sciences, Kermanshah 6734667149, Iran; ^2^Department of Pharmacognosy and Biotechnology, School of Pharmacy, Kermanshah University of Medical Sciences, Kermanshah 6734667149, Iran; ^3^Students Research Committee, School of Pharmacy, Kermanshah University of Medical Sciences, Kermanshah 6734667149, Iran; ^4^Medical Biology Research Center, Kermanshah University of Medical Sciences, Kermanshah 6734667149, Iran

## Abstract

Natural products are excellent resources for finding lead structures for the development of chemotherapeutic agents. Coumarins are a class of natural compounds found in a variety of plants. In this study, we evaluated the cytotoxic potential of coumarins isolated from *Prangos ferulacea* (L.) Lindl. in PC3, SKNMC, and H1299 (p53 null) human carcinoma cell lines. Osthole proved to be an outstanding potent cytotoxic agent especially against PC3 cells. Isoimperatorin exhibited moderate inhibitory effect against SKNMC and PC3 cell lines. Oxypeucedanin and braylin did not display any cytotoxic activity. In the next set of experiments, the apoptotic potentials of osthole and isoimperatorin were investigated. Induction of apoptosis by isoimperatorin was accompanied by an increase in activation of caspase-3, -8, and -9 in SKNMC cells and caspase-3 and -9 in PC3 cells. Moreover, isoimperatorin induced apoptosis by upregulating Bax and Smac/DIABLO genes in PC3 and SKNMC cells. Osthole induced apoptosis by downregulating antiapoptotic Bcl-2 in only PC3 cells and upregulating the proapoptotic genes Bax and Smac/DIABLO in PC3, SKNMC, and H1299 cells. The effects of osthole on H1299 cells are important because the loss of p53 has been associated with poor clinical prognosis in cancer treatment.

## 1. Introduction

New anticancer therapeutics are necessary to minimize various complications endured by cancer patients. The most common tumors frequently resist treatment with a significant number of commercially available anticancer drugs; therefore, the growing demand for developing more effective anticancer agents continues to exist. Higher plants provide a good source of clinically relevant compounds with anticancer properties. These classes of compounds include terpenoids, alkaloids, and various phenolic compounds such as coumarins [1]. 

Coumarins (2*H*-1-benzopyran-2-one) represent a class of phenolic compounds isolated from plants; the structural characteristic of coumarins depicts a framework consisting of fused benzene and *α*-pyrone ring systems [2]. Coumarins are well-known for their potential pharmacological activities: anti-inflammatory, antioxidant [3], antiviral [4], antimicrobial [5], and anticancer [6].

Moreover, scientists have reported potent cytotoxic activity of a number of coumarin compounds in various cancer cell lines including A549 (lung), A375 (skin), MCF-7 (breast), HSCs (liver), and HL-60 (leukemia) [7–11].

In this study, we have used* Prangos ferulacea *(L.) Lindl. as a source for coumarin isolation because Apiaceous plants provide an attractive resource for obtaining furanocoumarins [12, 13]. The plant,* P. ferulacea* commonly grows in the Mediterranean and Middle-East regions including Iran [14]; the antioxidant, antibacterial [15], and antispasmodic effects [16, 17] of this plant have been reported, and also it is widely used as provender for mutton [12].* P. ferulacea* provides a major source of coumarins compounds such as osthole, isoimperatorin, and oxypeucedanin and coumarins such as gosferol, pranferol, oxypeucedanin methnolate, oxypeucedanin hydrate, and psoralen [18, 19] occur in minor amounts. This is the first report describing the cytotoxicity of braylin (6-methoxyseselin) isolated from this plant. This compound belongs to the group of angular furanocoumarins, which are rare in nature. Braylin has shown to exhibit vasorelaxant [20] and moderate anti-HIV effect [21].

Further research is essential to gain better understanding of the anticancer activity of coumarins. The present study compares the cytotoxic and apoptotic inducing effects of four coumarin compounds, namely, osthole, isoimperatorin, oxypeucedanin, and braylin (isolated from* P. ferulacea*) on H1299 (human non-small cell lung carcinoma), SKNMC (human neuroblastoma), and PC3 (human prostate cancer) cell lines. The findings presented herein constitute the first report to demonstrate the proapoptotic activity of the coumarins osthole and isoimperatorin in SKNMC and PC3 cells. We have also demonstrated that osthole induced apoptosis in H1299 cells through a mechanism independent of tumor suppressing action of p53 protein because H1299 cell line is devoid of genes encoding p53 protein.

## 2. Materials and Methods

### 2.1. Plant Material and Coumarins Isolation

**Figure 1:**
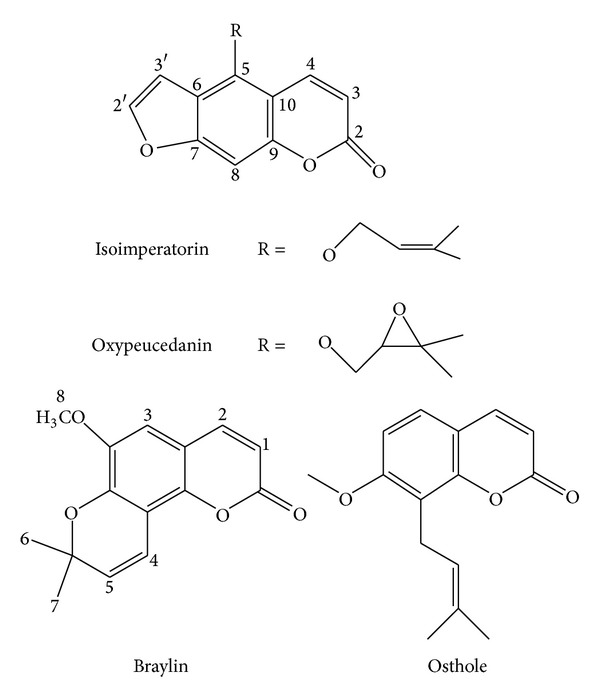
Chemical structures of isolated coumarins.

Plant material gathering, identification and extraction, and isolation of osthole, isoimperatorin, and oxypeucedanin (Figure 1) were performed as previously reported [22]. Braylin was purified by using HPLC (petroleum ether (P) : ethyl acetate (E),  6 : 94) separation from a fraction obtained from an open column chromatography (P : E, 10 : 90) of defatted acetone extract. Structures of all compounds were elucidated by using ^1^H-NMR, ^13^C-NMR, and Mass spectra, comparing to literature [12, 22–24].

### 2.2. Cell Culture Conditions

H1299 (human nonsmall cell lung carcinoma) cell line was a kind gift from Prof. G. Storm. The cells have a homozygous partial deletion of the p53 protein and lack p53 protein expression. SKNMC, human neuroblastoma cell line, and PC3, human prostate cancer cell line, were obtained from Pasteur Institute (Tehran, Iran). PC3 was established in 1979 from bone metastasis of grade IV of prostate cancer in a 62-year-old Caucasian male. This cell line is useful to assess prostatic cancer cells response to chemotherapeutic agents [25]. SKMNC, is a neuroepithelioma cell line derived from a metastatic supraorbital human brain tumor [26]. The cells were cultured in Dulbecco's modified Eagle's medium (DMEM-F12) with 5% (v/v) fetal bovine serum, 100 Uml^−1^ penicillin, and 100 mgml^−1^ streptomycin. The medium was changed every 2-3 days and subcultured when the cell population density reached to 70–80% confluence. 

### 2.3. Viability Assay

The cytotoxic effects of isolated coumarins were determined against cell lines by a colorimetric assay using 3-(4,5-dimethylthiazol-2-yl)-2, 5-diphenyltetrazolium bromide (MTT) and were compared with the untreated control. Cells were plated onto 96-well plates at a density of 2.0 × 10^4^ cells/well and in a volume of 200 *μ*L. Stock solutions of isolated coumarins were prepared in dimethyl sulfoxide (DMSO). The final concentration of the vehicle in the medium was always 0.5%. One day after seeding, 2 *μ*L of the DMSO containing coumarins at different concentrations was added to each well. At appropriate time intervals, the medium was removed and replaced by 100 *μ*L of 0.5 mg/mL of MTT in growth medium and then the plates were transferred to a 37°C incubator for 3-4 h. Supernatants were removed and the reduced MTT dye was solubilized with DMSO (100 *μ*L/well). Absorbance was determined on an ELISA plate reader (Biotek, H1M) with a test wavelength of 570 nm and a reference wavelength of 630 nm to obtain sample signal (OD570–OD630). Percentage of proliferation was calculated using the following formula: percent of control proliferation = (OD test/OD control) × 100. IC_50_ values were calculated by plotting the log⁡⁡10 of the percentage of proliferation versus drug concentration.

### 2.4. Detection of Caspase-3, -8, and -9 Activation

Caspase 3, 8, and 9 assays were carried out using the sigma colorimetric caspase kit. This assay was based on the ability of the active enzyme to cleave the chromophore from the enzyme substrates, Ac-DEVD-pNA (for caspase-3), Ac-IETD-pNA (for caspase-8), and Ac-LEHD-pNA (for caspase-9), in equal amount of cells protein. The cells (5 × 10^5^) were harvested and lysed in 70 *μ*L of the cell lysis buffer included with the kit, and protein concentrations were equalized for each condition. Subsequently, 10 *μ*L of cell lysate was combined with an equal amount of substrate reaction buffer containing caspase-3, -8, and -9 colorimetric substrates. This mixture was incubated for 2 h at 37°C, and then absorbance was measured with a plate reader (BioTek, H1M).

### 2.5. Real Time RT-PCR Analysis of Apoptosis-Related Gene Expression

Total RNA from SKNMC, PC3, and H1299 cells pretreated with IC_50_ concentration of coumarins were extracted using high pure isolation kit (Roche, Mannheim, Germany) according to the manufacture instructions. Quality and quantity of total RNA were assessed by spectrophotometer (NanoDrop 2000, USA) and samples were stored at −80°C until use. The primer sequences used for PCR were *β*-actin: 5′-TCATGAAGTGTGTGACGTGGACATC-3 (forward) and 5′-CAGGAGGAGCAATGATCTTGATCT-3′ (reverse); Bcl-2: 5′-ATCGCCCTGTGGATGACTGAG-3′ (forward) and 5′-GACCCAGGAGAAATCAAACAGAGG-3′ (reverse); Bax: 5′-GGACGAACTGGACAGTAACATCG-3′ (forward) and 5′-GCAAAGTAGAAAAGGGCGACAAC-3′ (reverse); and Smac/DIABLO: 5′-AGCTGGAAACCACTTGGATG-3′ (forward) and 5′-CCAGCTTGGTTTCTGCTTT-3′ (reverse) [27, 28]. The performances of all primer pairs were tested by primer concentration to determine the optimal reaction conditions. Thermal cycler conditions were 15 min at 50°C for cDNA synthesis, 10 min at 95°C followed by 40 cycles of 15 s at 95°C to denature the DNA, and 45 s at 60°C to anneal and extend the template. Melting curve analysis was performed to ascertain specificity by continuous acquisition from 65°C to 95°C with a temperature transient rate of 0.1°C/S. All reactions were performed in triplicate in a Corbett system (Australia). The value obtained for the target gene expression was normalized to *β*-actin and analyzed by the relative gene expression −ΔΔCT method, where −ΔΔCT = (CT target − CT *β*-actin) unknown − (CT target − CT *β*-actin) calibrator.

### 2.6. Statistical Analysis

Each experiment was performed at least three times, and the results were presented as mean ± S.E.M. One-way analysis of variance (ANOVA) followed by Turkey's test was used to compare the difference between means. A probability value of *P* < 0.05 was considered to be statistically significant.

## 3. Results

### 3.1. Inhibition of Cell Viability

**Figure 2:**
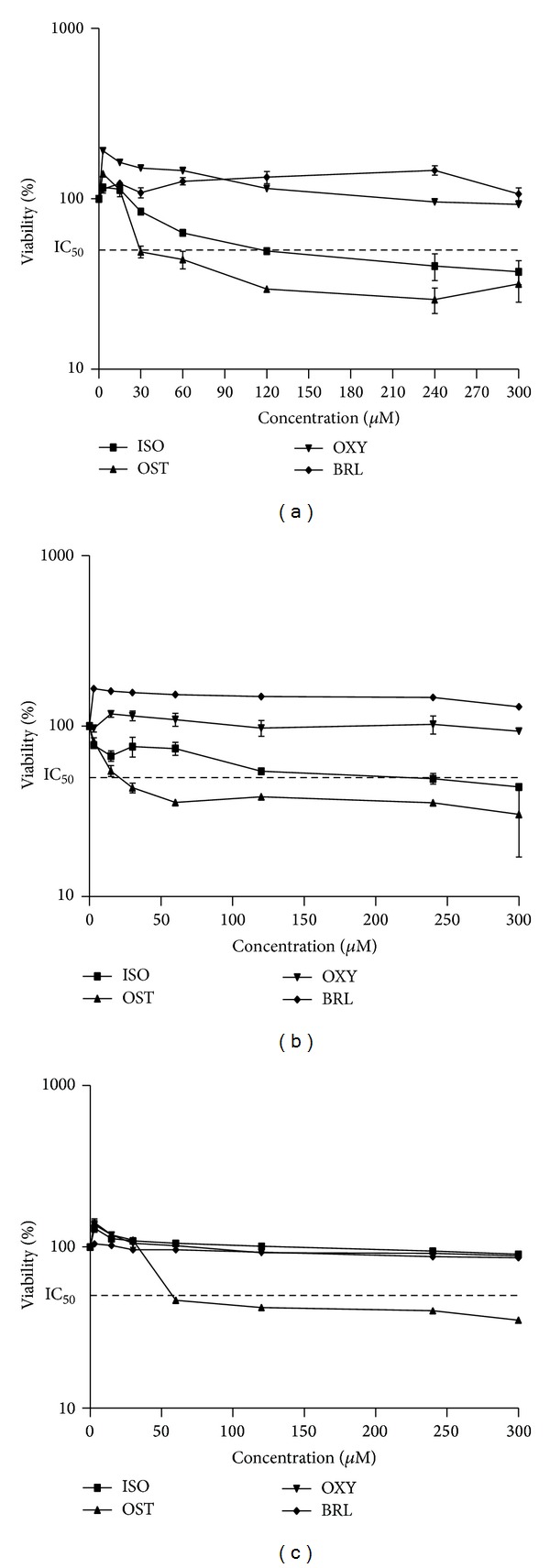
Cytotoxic effects of four isolated coumarins in (a) SKNMC, (b) PC3, and (c) H1299 cancer cells. The cells were incubated with at different concentrations of coumarins for 24 h. The cell proliferation inhibition was determined by MTT assay as described under materials and methods. Data are presented as mean ± S.E.M (*n* = 3).

The potency of isolated coumarins to induce cell death was determined on SKNMC, PC3, and H1299 cell lines under MTT method. The results indicated that the cell proliferation was inhibited in the order of osthole > isoimperatorin > oxypeucedanin ≥ braylin in three cell lines. As shown in Figures 2(a)–2(c), exposure to osthole for 24 h resulted in a concentration dependent decrease in cell viability, with approximate IC_50_ of 28.81 ± 0.79 *μ*M, 20.08 ± 2.1 *μ*M, and 58.43 ± 4.08 in SKNMC, PC3, and H1299 cells, respectively. Isoimperatorin possessed a moderate inhibitory effect against SKNMC (IC_50_ = 182 ± 10.91 *μ*M) and PC3 (IC_50_ = 119.4 ± 8.65 *μ*M) cell lines and had no effect against H1299 cell line. On the contrary, toxicity was not observed after exposure to oxypeucedanin and braylin at the concentrations up to 300 *μ*M in the above mentioned cell lines (Table 1). The results indicated that osthole and isoimperatorin have the highest antiproliferative effect towards PC3, SKNMC, and H1299 cell lines, respectively. Therefore, they were selected and used for further studies.

### 3.2. Effects of Osthole and Isoimperatorin on Caspase-3, -8, and -9 Activity

**Figure 3:**
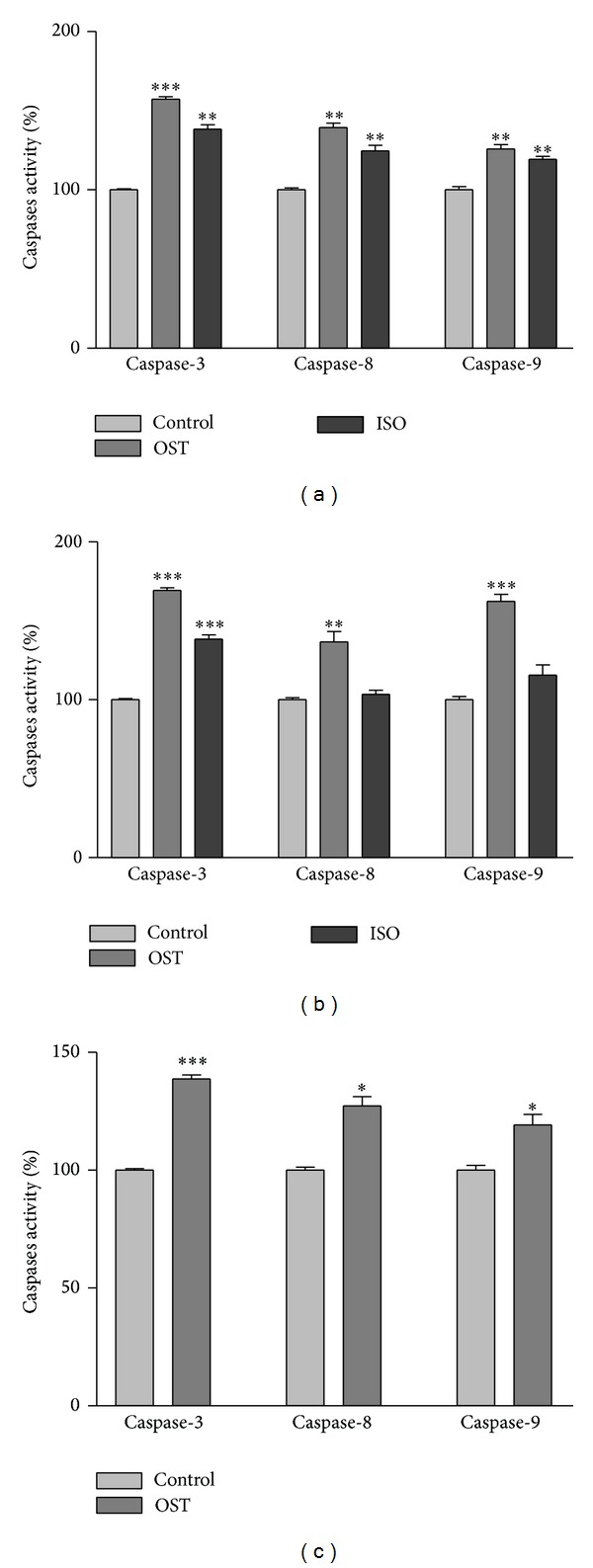
Involvement of activation of caspases in the induction of apoptosis on (a) SKNMC, (b) PC3, and (c) H1299 human cancer cells. Cells were incubated with IC_50_ concentration of the indicated compounds and harvested at 24 h and cell lysates were assayed using microplate reader for activation caspases. Significant differences were compared with the control. Data are presented as mean ± S.E.M. *P < 0.05, **P < 0.01, and ***P < 0.001 versus control.

Activation of caspase cascade is critical in the initiation of apoptosis in various biological systems [29]. Therefore, for improvement of MTT results and also characterizing the type of cell death involved in our experiments, the activity of caspases was examined. A member of this family, caspase-3, has been identified as being a key mediator of apoptosis [30]. The obtained results showed that 24 h treatment with IC_50_ concentration of osthole and isoimperatorin increased caspase-3 activation in human carcinoma cell lines. To determine which apoptotic pathway is activated by osthole and isoimperatorin, we evaluated the activation of caspase-8 and -9, the apical proteases in extrinsic and intrinsic pathways, respectively [31]. Osthole was able to increase caspase-8 and -9 in H1299, PC3, and SKNMC cells, thus implying that osthole induces apoptosis in these cell lines through both intrinsic and extrinsic pathways. Like osthole, isoimperatorin increased both caspases activities in SKNMC cells. While in the PC-3 cells, isoimperatorin could only increase caspase-9 activity (Figure 3).

### 3.3. Effect of Osthole and Isoimperatorin on Some Critical Genes Involved in Apoptosis

**Figure 4:**
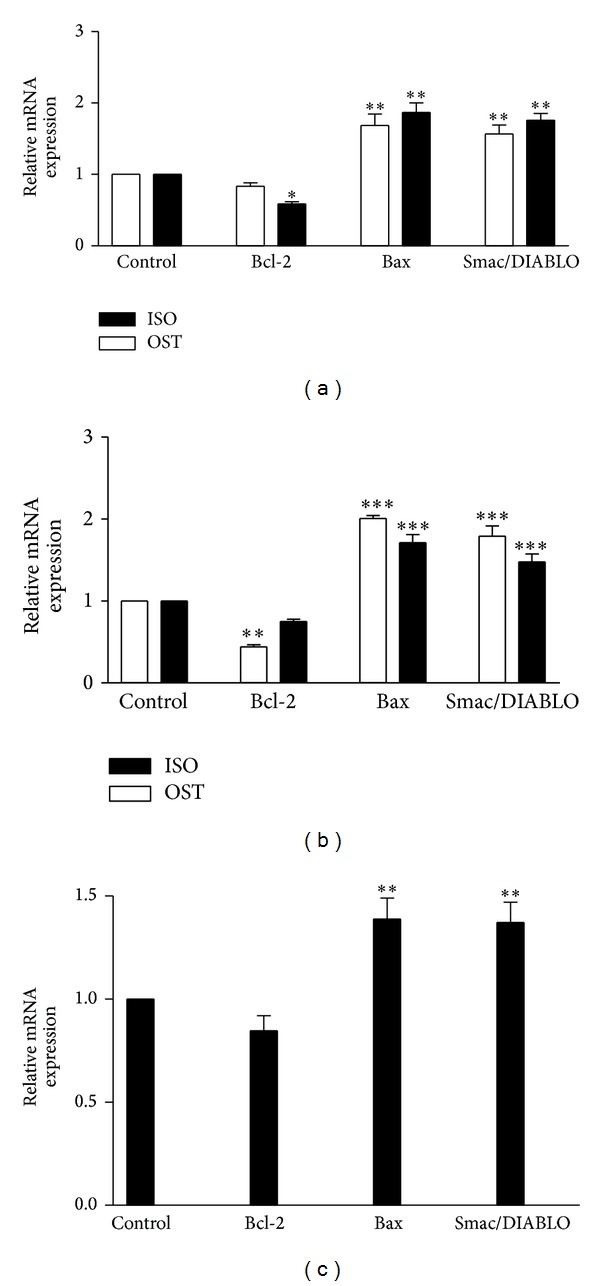
The effect of coumarins on expression of apoptotic-related genes on (a) SKNMC, (b) PC3, and (c) H1299 human cancer cells. Normalization relative to b-actin was performed. Levels of mRNA are expressed relative to control in the mean ± S.E.M values derived from three independent experiments. *P < 0.05, **P < 0.01, and ***P < 0.001 versus control.

The mitochondria are an integral part of the apoptotic machinery; therefore, we analyzed the most important proteins involved in mitochondrial pathway of apoptosis (Bax and Bcl-2) [32]. We found that osthole was able to decrease significantly Bcl-2 mRNA expression in only PC-3 cell line. However, the Bax mRNA expression decreased significantly upon treatment with osthole in three cell lines. Next, the mRNA expression of Smac/DIABLO was measured. It is a protein released from mitochondria in response to apoptotic stimuli. Smac/DIABLO antagonizes inhibitor of apoptosis proteins (IAPs) to relieve their inhibitory effects on caspases [27]. The obtained results demonstrated that 24 h treatment with osthole increased significantly Smac/DIABLO in the level of mRNA expression in all cell lines. Furthermore, real time RT-PCR analysis clearly shows a significant reduction in the expression level of Bcl-2 after 24 h treatment with isoimperatorin in SKNMC cells. Moreover, induction of apoptosis by isoimperatorin was accompanied by increase in mRNA levels of proapoptotic Bax and Smac/DIABLO genes in PC3 and SKNMC cells (Figure 4).

## 4. Discussion

Herbs and herbal extracts offer a wide variety of phytochemicals with diverse biological functions. Among these phytochemicals, coumarins play a significant role in plant biochemistry and physiology including antioxidants, enzyme inhibitors, and precursors of toxic substances in biochemical reactions involving different cellular systems [6]. Among their diverse biological properties, the antitumor activities and antiproliferative effects have been extensively studied and reported [33].

In the current study, the following four coumarins were isolated from* P. ferulacea*: osthole, isoimperatorin, oxypeucedanin, and braylin. Among the isolates, osthole and isoimperatorin showed the highest inhibitory potency against the growth of human carcinoma cell lines whereas oxypeucedanin and braylin failed to exhibit any cytotoxic effects. The present findings corroborate the findings reported by Yang et al., who evaluated cytotoxicity of coumarins isolated from fruits of* Cnidium monnieri* on leukemia cell lines. Their results revealed that, among osthole, imperatorin, bergapten, isopimpinellin, and xanthotoxin, osthole showed the strongest cytotoxic activity in tumor cell lines [34]. In our study, PC3 cells showed the highest sensitivity toward osthole and isoimperatorin. This result supports previous literature reports, which indicated that osthole (100 *μ*M) had a weak but significant antiproliferative activity against hormone independent PC3 and DU145 human prostate cancer cell lines [35]. In addition, there is a report showing antiproliferative effects of isoimperatorin on DU145 cell line [36]. We also found that H1299 cells were more susceptible to treatment with osthole but isoimperatorin had no antiproliferative effect on this cell line.

The apoptosis-inducing capacity rather than necrosis induction is preferably considered as a key feature of a potential antitumour drug. Accordingly, in the next set of experiments we investigated the apoptosis-inducing potentials of the cytotoxic agents. The Bcl-2 family proteins have emerged as a key component in regulating apoptosis; they either inhibit or promote cell death [28]. Bax and Bcl-2, the two important members of this family, influence the permeability of mitochondrial membrane. Cell survival in the early phases of apoptotic signaling cascade depends mostly on the balance between proapoptotic and antiapoptotic proteins of the Bcl-2 family [31]. Permeability of mitochondrial membrane allows the release of soluble molecules from the outer space of the mitochondria to the cytosol. One of these representative molecules which is called Smac/DIABLO activates a cascade of caspases in the cytosol [27]. The activation of caspases triggers the release of cytochrome C leading to the deactivation of apoptosis inhibitory proteins belonging to the (IAP) family [29]. Our results showed that osthole was able to increase caspase-3, -8, and -9 activities in H1299, PC3, and SKNMC cells. Moreover, osthole induced apoptosis by downregulating antiapoptotic Bcl-2 in PC3 cells and upregulating proapoptotic genes Bax and Smac/DIABLO in PC3, SKNMC, and H1299 cells thereby, implying that osthole induces apoptosis in these cells via both mitochondrial and extrinsic pathways. As mentioned before, H1299 cells lack the expression of p53 protein. The p53 tumor suppressor proteins play a pivotal role in initiating apoptosis by sensing different intrinsic and extrinsic stresses. Defect in p53 function alone leads to phenotypic resistance resulting in chemotherapeutic failure of cancer treatment. The lack of p53 expression in H1299 may account for its higher resistance to cell death in comparison with other carcinoma cells [37].

Our results indicated that osthole caused an increase in expression of proapoptotic protein Bax via a p53 independent pathway and the increase in Bax expression induced the initiation of apoptotic cell death. The potential effect of osthole on H1299 cells is very promising as this compound can be applied to treat different types of tumors that display deregulated tumor suppression pathways under p53 control. However, further studies are needed to determine the exact molecular mechanisms induced by osthole in human nonsmall cell lung cancer carcinoma. In accordance with this finding, it has been previously reported that 7,8-dihydroxy-4-methylcoumarin (DHMC) mediates apoptosis in human leukemic HL-60 and U-937 cells via mechanisms independent of p53 activity [38]. Additionally, another study has described the p53 independent cytotoxic effects of coumarin and hydroxyl coumarins on squamous carcinoma cell lines [37]. We also found that isoimperatorin induces apoptosis by upregulating proapoptotic Bax and Smac/DIABLCO in PC3 and SKNMC cells. Moreover, isoimperatorin mediated apoptosis was accompanied by an increase in activation of caspase-3, -8, and -9 in SKNMC cells and caspase-3 and -9 in PC3 cells. Therefore, it appears that apoptosis induced by isoimperatorin occurred via an intrinsic pathway in PC3 cells.

It is difficult to render a structure activity relationship among the coumarin analogues tested from* P. ferulacea *because they come from different class types. For example, osthole is a prenylated coumarin, isoimperatorin and oxypeucedanin are linear furanocoumarins, and braylin is an angular pyranocoumarin. However, Riveiro et al. proved that alkoxy residue could potentiate the antiproliferative effects of coumarins [39]. Although the tested coumarins were devoid of catechol moiety, which could have contributed to the growth inhibitory activity [40], it is highly likely that the presence of isoprenoid residue in osthole and isoimperatorin has contributed to various pharmacological interactions, which gave rise to the proapoptotic activity [41]. Important coumarin apoptotic features such as free catechol and isoprenoid moieties are absent in braylin and oxypeucedanin. Oxidation or cyclization of isoprenoid in isoimperatorin and osthole, respectively, resulted in inactive oxypeucedanin and braylin.

In conclusion, osthole showed the best cytotoxic activity among the four coumarin compounds tested. Osthole can induce apoptosis in PC3, H1299, and SKNMC cells at low micromolar concentrations. Therefore, osthole can be considered as a promising lead in cancer drug discovery and development.

## Figures and Tables

**Table 1:** Cytotoxic activity of coumarins isolated from *Prangos ferulacea *against human cancer cell lines.

	SKNMC		PC3		H1299	
	IC_50_ (*μ*M)	MAE (%)	IC_50_ (*μ*M)	MAE (%)	IC_50_ (*μ*M)	MAE (%)
Osthole	28.81 ± 0.79	69.65 (5.93)	20.1 ± 2.1	70.38 ± 2.6	58.43 ± 2.15	52.18 ± 10.94
Isoimperatorin	182 ± 10.91	63.38 ± (10.94)	119.4 ± 8.65	55.14 ± 1.74	>300	13.89 ± 3.01
Oxypeucedanin	> 300	<10	>300	<10	>300	<10
Braylin	>300	<10	>300	<10	>300	<10

MAE: maximum antiprolifrative effect.
